# Integrated Assessment of Percentage α2,3-Linked Sialylated Prostate-specific Antigen and the Prostate Health Index with Magnetic Resonance Imaging for Detection of Clinically Significant Prostate Cancer

**DOI:** 10.1016/j.euros.2025.12.012

**Published:** 2025-12-26

**Authors:** Takuya Oishi, Tohru Yoneyama, Yuki Miura, Tomoko Hamaya, Hirotake Kodama, Takuma Narita, Jotaro Mikami, Naoki Fujita, Teppei Okamoto, Hayato Yamamoto, Atsushi Imai, Chikara Ohyama, Shingo Hatakeyama

**Affiliations:** a Department of Urology, Hirosaki University Graduate School of Medicine, Hirosaki, Japan; b Department of Glycotechnology, Hirosaki University Graduate School of Medicine, Hirosaki, Japan; c Department of Advanced Transplant and Regenerative Medicine, Hirosaki University Graduate School of Medicine, Hirosaki, Japan

**Keywords:** Prostate cancer, Prostate Health Index, α2,3-Linked sialylated prostate-specific antigen

## Abstract

**Background and objective:**

The diagnostic roles of serum percentage α2,3-linked sialylated prostate-specific antigen (S2,3PSA%) and the Prostate Health Index (PHI) in predicting clinically significant prostate cancer (csPC) remain unclear in the context of magnetic resonance imaging (MRI)-guided biopsy. Our aim was to evaluate the associations of S2,3PSA% and PHI with csPC and to develop an internally validated nomogram that integrates these biomarkers with prostate MRI.

**Methods:**

This retrospective single-center study included 248 consecutive men who underwent both S2,3PSA% and PHI testing, followed by MRI-ultrasound fusion targeted biopsy between October 2018 and July 2025. We used multivariable logistic regression models to identify predictors of csPC (Gleason ≥7). Internal validation was conducted with 1000 bootstrap resamples to estimate optimism and calculate the optimism-corrected area under the receiver operating characteristic curve (AUC), calibration slope, and Brier score. Decision curve analysis (DCA) was used to assess the clinical net benefit of the nomogram.

**Key findings and limitations:**

Among 248 patients, csPC was detected in 111 (45%). Age, S2,3PSA%, and Prostate Imaging-Reporting and Data System (PI-RADS) score were independent predictors of csPC. The nomogram achieved an apparent AUC of 0.857. Internal bootstrap validation yielded an optimism-corrected AUC of 0.783, calibration slope of 0.911, and Brier score of 0.139, which confirm good model discrimination and calibration. DCA demonstrated a clear net benefit for the nomogram across clinically relevant threshold probabilities. The single-center design and lack of external validation limit the generalizability of our results.

**Conclusions and clinical implications:**

Integration of S2,3PSA%, PHI, and PI-RADS scores provides incremental diagnostic utility for csPC detection. Internally validated models suggested better discrimination on integration of these biomarkers with MRI. However, these findings are exploratory and require external validation.

**Patient summary:**

We found that combining blood biomarkers called S2,3PSA% and the Prostate Health Index with MRI (magnetic resonance imaging) scan findings improved prediction of whether a patient has prostate cancer. Larger studies are needed to confirm our results.

## Take Home Message

α2,3-Linked sialylated prostate-specific antigen (PSA), a cancer-associated PSA glycosylation biomarker, and the Prostate Health Index outperformed PSA in identifying clinically significant prostate cancer in a single-center cohort. When combined with magnetic resonance imaging findings, these markers modestly improved risk stratification. Further multicenter validation is warranted before clinical application.

## 1 Introduction

Prostate-specific antigen (PSA) testing has long been the cornerstone of prostate cancer (PC) detection [1], [2], [3], [4], [5], [6]. However, its limited specificity leads to unnecessary prostate biopsies and the overdiagnosis of indolent disease [7], [8], [9], [10], [11], [12], [13]. To overcome these limitations, several PSA derivatives and composite indices, such as the Prostate Health Index (PHI), have been introduced and shown to improve the discrimination of clinically significant PC (csPC; Gleason ≥7 or International Society of Urological Pathology grade group ≥2) [12], [13], [14], [15], [16], [17], [18]. Despite these advances, the accuracy of current biomarkers remains suboptimal, particularly in populations with a high prevalence of indolent tumors.

Alterations in glycosylation are a well-recognized hallmark of cancer, and recent developments have facilitated the measurement of cancer-associated glycoforms of PSA. Among these, percentage α2,3-linked sialylated PSA (S2,3PSA%) has emerged as a promising biomarker, with preliminary studies suggesting its value in distinguishing csPC from benign disease [19], [20], [21], [22], [23], [24], [25], [26]. Nevertheless, clinical validation of S2,3PSA% in biopsy cohorts remains limited, and its role in combination with established biomarkers such as PHI has not been fully clarified.

Magnetic resonance imaging (MRI), and specifically the Prostate Imaging-Reporting and Data System (PI-RADS), has improved risk stratification for csPC, yet the predictive performance of MRI alone is insufficient [12], [13], [27]. There is a growing need for integrative models that combine blood-based biomarkers with imaging to better guide biopsy decisions [28], [29], [30], [31], [32]. Therefore, the aim of our study was to validate the clinical utility of S2,3PSA% and PHI for detecting csPC. In addition, we assessed their value in risk modeling with MRI to guide biopsy decision-making.

## 2 Patients and methods

### 2.1 Study design

This study involved retrospective analysis of prospectively collected data. The study was conducted in accordance with the Declaration of Helsinki and was approved by the ethics committee of the Hirosaki University School of Medicine (2020-212-1 and 2019-099-3). Written informed consent was waived because of the retrospective design, with an opt-out approach that provided patients with the opportunity to decline participation.

### 2.2 Study population

**Fig. 1:**
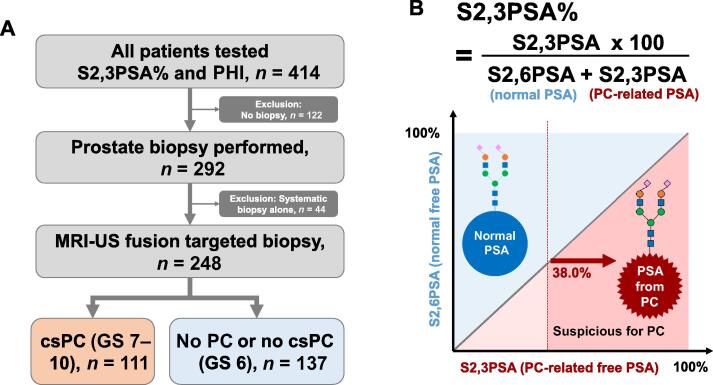
Study flow and definition of S2,3PSA%. (A) Flow diagram of patient selection. Among 414 patients who underwent PHI and S2,3PSA% testing, 122 were excluded because of no biopsy and 44 because of systematic biopsy alone, leaving 248 patients who underwent MRI-US fusion targeted biopsy for analysis. Of these, 111 were diagnosed with csPC (GS ≥7) and 137 had no PC or clinically insignificant PC (GS 6). (B) Definition of S2,3PSA%. S2,6PSA represents the normal PSA glycoform, and S2,3PSA represents the cancer-related PSA glycoform. A cutoff value of 38.0% was applied to distinguish suspicious PC cases. MRI = magnetic resonance imaging; US = ultrasound; PHI = Prostate Health Index; PSA = prostate-specific antigen; S2,3PSA% = percentage α2,3-linked sialylated PSA; S2,6PSA = α2,6-linked sialylated PSA; PC = prostate cancer; csPC = clinically significant PC; GS = Gleason score.

We retrospectively reviewed data for 465 men who underwent both S2,3PSA% and PHI testing before prostate biopsy at Hirosaki University Hospital between October 2018 and July 2025. Patients were referred after initial PSA testing and MRI. At enrollment, S2,3PSA%, and PHI were measured for research purposes; thus, S2,3PSA% and PHI testing was not part of routine care, but were performed under the study protocol. Patients with PSA ≥4.0 ng/ml who underwent S2,3PSA%, PHI, and MRI before MRI-ultrasound (US) fusion biopsy were included. Those lacking any test, under active surveillance, or with prior transurethral resection of the prostate were excluded. Men with a low-risk profile (low PHI, low S2,3PSA%, negative MRI) opted for surveillance after shared decision-making, whereas some with normal biomarkers but suspicious MRI (PI-RADS 4–5) proceeded to biopsy. Patients who underwent systematic biopsy alone (*n* = 44) were excluded, as MRI-US fusion targeted biopsy served as the diagnostic reference standard in this study (Fig. 1A). The final cohort consisted of 248 men who underwent multiparametric MRI followed by MRI-US fusion targeted biopsy with additional systematic cores. Clinical data including age, PSA, prostate volume, digital rectal examination (DRE) findings, prostate volume (in ml), history of previous prostate biopsy, Gleason score (GS), and clinical T stage were extracted from medical records.

### 2.3 Biomarker measurements

Serum S2,3PSA% was measured using a lectin-antibody immunoassay on a microTAS Wako i50 analyzer (FUJIFILM Wako Pure Chemical Corporation, Osaka, Japan), which specifically detects α2,3-linked sialylated PSA isoforms. S2,3PSA% was calculated as the ratio of cancer-associated PSA (S2,3PSA) to the sum of S2,3PSA and the normal glycoform (S2,6PSA) [24]. This index allows more accurate detection of PC in comparison to total PSA alone, and a predefined cutoff of ≥38.0% was applied to define cancer suspicion (Fig. 1B). PHI was calculated as (p2PSA/free PSA) × √(total PSA). PHI measurement was outsourced to BML (Tokyo, Japan), a certified clinical laboratory, and performed according to the manufacturer’s instructions. All assays were conducted under standardized laboratory protocols [12]. Insurance cover was approved for S2,3PSA% in February 2024 and for PHI in November 2021. S2,3PSA% was previously measured for research, but testing is now routine for biopsy candidates. The PHI test was conducted on stored serum, and both markers were evaluated together for combined diagnostic value. We used previously reported cutoffs (PHI 27.2; S2,3PSA% 38.0%) [12], [25].

### 2.4 MRI and biopsy procedures

All patients underwent multiparametric MRI using a 1.5-T or 3-T scanner. Lesions were scored according to PI-RADS v2.1. MRI scans were acquired using different scanners across institutions but were centrally reviewed by a single prostate MRI specialist to ensure consistent PI-RADS scoring and minimize interobserver variability. Targeted biopsies of PI-RADS ≥3 lesions were performed using a BioJet 3D-MRI/TRUS Image Fusion Prostate Biopsy System (D&K Technologies GmbH, Barum, Germany). Two cores were obtained from each target lesion, and a ten-core systematic biopsy was also performed in all patients. Biopsy specimens were reviewed by dedicated genitourinary pathologists. csPC was defined as Gleason score ≥7 (grade group ≥2).

### 2.5 Outcomes

The primary outcome was the diagnostic accuracy of S2,3PSA% and PHI for csPC detection. Secondary outcomes included the performance of a multivariable risk model incorporating age, PHI, S2,3PSA%, and PI-RADS parameters.

### 2.6 Statistical analysis

Continuous variables are summarized as the median with interquartile range (IQR) and were compared using Mann-Whitney *U* tests, while categorical variables were compared using χ2 or Fisher’s exact tests. Receiver operating characteristic (ROC) analyses were performed to evaluate the discriminative ability of individual biomarkers. csPC detection rates were examined across combinations of biomarker positivity and PI-RADS categories. Associations between biomarkers and csPC were further assessed within each PI-RADS category using Mann-Whitney *U* tests. Logistic regression was used to identify independent predictors of csPC. A multivariable logistic regression model incorporating age, PHI, S2,3PSA%, and highest PI-RADS score (categorical: 3, 4, and 5) as predictors was fitted. Nomogram-based probability estimates were derived from the multivariable model, with predicted csPC probabilities plotted across S2,3PSA% and PHI levels for PI-RADS 3–5 lesions. Internal validation was performed using 1000 bootstrap resamples to estimate model optimism and obtain optimism-corrected results for the area under the ROC curve (AUC), calibration slope/intercept, and Brier score. Model calibration was evaluated using calibration plots. Clinical utility was assessed using decision curve analysis. Statistical significance was defined as two-sided *p* < 0.05. All analyses were conducted using BellCurve for Excel version 4.07 (Social Survey Research Information, Tokyo, Japan) and GraphPad Prism version 7 (GraphPad Software, La Jolla, CA, USA).

## 3 Results

### 3.1 Patient characteristics

Of 414 men with S2,3PSA% and PHI testing, 122 who did not undergo biopsy and 44 who received systematic biopsy alone were excluded, leaving 248 men who underwent MRI-US fusion biopsy for analysis (Fig. 1A). Overall, 111 (45%) had csPC (GS 7–10) and 137 (55%) had non-csPC or benign findings. The group with csPC was older (72 vs 68 yr), had higher PSA, PHI, and S2,3PSA% values, lower prostate volume (30 vs 40 ml), and higher PI-RADS scores (all *p* < 0.001; Table 1).

**Table 1:** Patient characteristics

Parameter	GS 7–10	GS 6	*p* value
Patients (*n*)	111	137	
Median age, yr (IQR)	72 (69–76)	68 (64–74)	<0.001
Median PSA, ng/ml (IQR)	10.5 (7.7–16.3)	8.1 (5.0–12.4)	<0.001
PSA >10 ng/ml, *n* (%)	59 (53)	51 (37)	0.015
Median S2,3PSA%, (IQR)	51.3 (43.5–59.0)	42.2 (35.1–47.9)	<0.001
Median Prostate Health Index score (IQR)	64.2 (43.8–98.8)	37.6 (28.6–50.6)	<0.001
Pathological outcome, *n* (%)			
No malignancy	–	110 (80)	
GS 6	–	27 (20)	
GS 7	54 (49)	–	
GS 8–10	57 (51)	–	
Clinical T stage, *n* (%) a			
cT1	68 (61)	–	
cT2	17 (15)	–	
cT3–4	26 (23)	–	
Highest PI-RADS score, *n* (%)			
3	9 (8.1)	49 (36)	<0.001
4	33 (30)	65 (47)	0.008
5	69 (62)	22 (16)	<0.001
Median prostate volume, ml (IQR)	33.0 (24.1–46.2)	49.1 (36.9–70.0)	<0.001
5α-Reductase inhibitor use, *n* (%)	12 (11)	18 (13)	0.4
Previous biopsy, *n* (%)	47 (42)	64 (47)	0.3

IQR = interquartile range; GS = Gleason score; PSA = prostate-specific antigen; PI-RADS = Prostate Imaging-Reporting and Data System; S2,3PSA% = percentage α2,3-linked sialylated PSA.

a Clinical T stage was not reported for the GS 6 cohort, as these cases did not have clinically significant prostate cancer.

### 3.2 Diagnostic performance

**Fig. 2:**
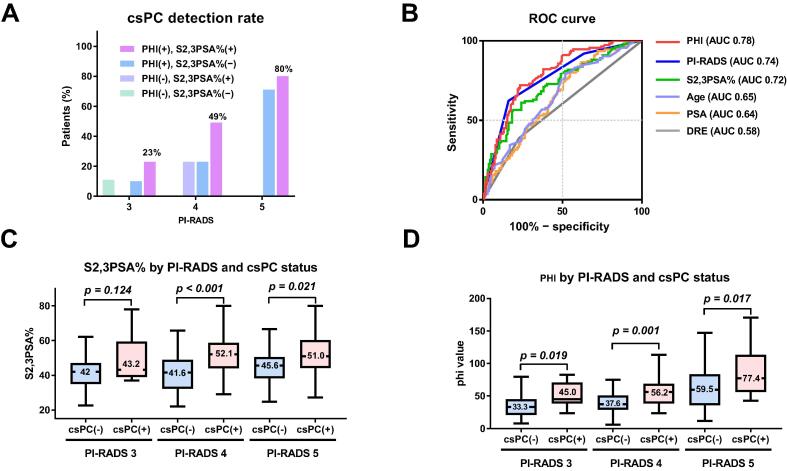
Diagnostic performance of PHI and S2,3PSA% for csPC detection. (A) csPC detection rates stratified by PI-RADS category and biomarker positivity. (B) ROC curves comparing the diagnostic performance of PHI, S2,3PSA%, PI-RADS, age, PSA, and DRE. (C) S2,3PSA% and (D) PHI values according to PI-RADS and csPC status. Both PHI and S2,3PSA% were significantly higher in the csPC group than in the group without csPC for PI-RADS 4–5, whereas differences were limited for PI-RADS 3. csPC = clinically significant prostate cancer; PHI = Prostate Health Index; S2,3PSA% = percentage α2,3-linked sialylated prostate-specific antigen; ROC = receiver operating characteristic; AUC = area under the ROC curve; PI-RADS = Prostate Imaging-Reporting and Data System; PSA = prostate-specific antigen; DRE = digital rectal examination.

The overall csPC detection rate was 45% (111/248 patients). The csPC detection rate was higher in the group of patients with positive results for both PHI (>27.2) and S2,3PSA% (>38.0%) in comparison to those with a positive result for only one marker or negative for both markers (Fig. 2A). Both biomarkers outperformed PSA (AUC 0.64) and DRE (AUC 0.58), and were comparable to PI-RADS (AUC 0.74; Fig. 2B). PHI (AUC 0.78) and S2,3PSA% (AUC 0.72) were significantly higher for csPC than for non-csPC for PI-RADS 4 (*p* < 0.001 and *p* = 0.001) and PI-RADS 5 (both *p* = 0.02), while only PHI remained significant for PI-RADS 3 (*p* = 0.02; Fig. 2C,D).

### 3.3 Multivariable analyses

According to the multivariable logistic regression model (Table 2), age (*p* < 0.001), S2,3PSA% (*p* < 0.001), and PI-RADS 5 (vs PI-RADS 3; *p* < 0.001) were independently associated with csPC. PHI (*p* = 0.11) and PI-RADS 4 (*p* = 0.065) showed borderline associations. Internal validation using 1000 bootstrap resamples demonstrated stable model performance, with an optimism-corrected AUC of 0.78, which confirms good discriminative ability. Calibration analysis revealed a near-ideal model fit (calibration slope 0.911) and satisfactory overall accuracy (Brier score 0.139). These results suggest that the nomogram provides reliable estimation of csPC probability with minimal overfitting.

**Table 2:** Multivariable logistic regression analysis for clinically significant prostate cancer

Parameter	Odds ratio (95% CI)	*p* value
Age	1.10 (1.04–1.15)	<0.001
Prostate Health Index	1.02 (1.00–1.02)	0.11
S2,3PSA%	1.03 (1.03–1.10)	<0.001
PI-RADS score		
PI-RADS 3	Reference	
PI-RADS 4	2.31 (0.95–5.60)	0.065
PI-RADS 5	10.7 (4.16–27.8)	<0.001

CI = confidence interval; PI-RADS = Prostate Imaging-Reporting and Data System; S2,3PSA% = percentage α2,3-linked sialylated prostate-specific antigen.

### 3.4 Nomogram development

**Fig. 3:**
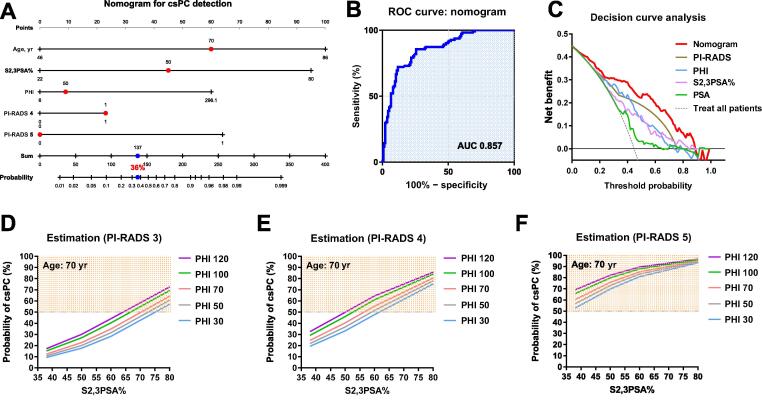
Development and performance of the nomogram for csPC detection. (A) Nomogram constructed using age, PHI, S2,3PSA%, and highest PI-RADS score for predicting csPC. (B) ROC curve for the nomogram (AUC 0.857). (C) Decision curve analysis (DCA) for csPC detection. (D–F) Estimation of csPC probability according to S2,3PSA% and PHI values for patients with (D) PI-RADS 3, (E) PI-RADS 4, and (F) PI-RADS 5 lesions. csPC = clinically significant prostate cancer; PHI = Prostate Health Index; S2,3PSA% = percentage α2,3-linked sialylated prostate-specific antigen; ROC = receiver operating characteristic; AUC = area under the ROC curve; PI-RADS = Prostate Imaging-Reporting and Data System.

A nomogram combining PHI, S2,3PSA%, and PI-RADS predicted csPC with excellent discrimination (AUC 0.857, Fig. 3A,B). Decision and net reduction analyses demonstrated a net reduction of 13.5–36.9 cases per 100 individuals at clinically relevant model thresholds of 0.3–0.6 (Table 3). The nomogram achieved the highest net clinical benefit across most threshold probabilities, outperforming PI-RADS, PHI, and S2,3PSA% alone (Fig. 3C). For PI-RADS 3–4 lesions (Fig. 3D–E), higher PHI and S2,3PSA% values were associated with a steeper increase in csPC probability, whereas for PI-RADS 5 lesions, csPC risk exceeded 50% irrespective of biomarker levels (Fig. 3F).

**Table 3:** Missed csPC cases and confusion matrix metrics by threshold probability for the nomogram

Model threshold	True positives	False negatives(missed csPC)	False positives	True negatives	csPC cases missed per 100
0.1	111	0	103	34	0
0.2	102	9	70	67	8.1
0.3	96	15	46	91	13.5
0.4	95	16	35	102	14.4
0.5	81	30	26	111	27.0
0.6	70	41	14	123	36.9
0.7	56	55	9	128	49.6
0.8	41	70	6	131	63.1
0.9	21	90	3	134	81.1

csPC = clinically significant prostate cancer.

## 4 Discussion

We validated the clinical utility of two blood-based biomarkers, S2,3PSA% and PHI, for detection of csPC in men undergoing MRI-targeted biopsy. Both biomarkers were independently associated with csPC and demonstrated higher diagnostic accuracy than PSA or DRE, with performance comparable to PI-RADS. These findings confirm the value of cancer-associated PSA glycosylation and composite PSA derivatives as adjuncts to imaging in PC diagnostics. The aim of our study was to compare diagnostic performance, and was not intended to show causal superiority. The higher net benefit of the nomogram reflects better discrimination, and not causality. Future multicenter validation with propensity-matched or penalized models may further clarify the added value of S2,3PSA%.

S2,3PSA% represents the proportion of α2,3-sialylated PSA—a glycoform mainly secreted by PC cells—relative to free PSA (S2,3PSA + S2,6PSA; Fig. 1B). As α2,6-sialylation predominates in benign tissue, elevated S2,3PSA% reflects tumor-associated glycosylation and thus confers higher specificity for csPC than total PSA.

Our findings build on prior studies by showing that combining S2,3PSA% with PHI improves risk stratification beyond MRI alone [24], [25]. In particular, biomarker positivity substantially increased the likelihood of csPC in PI-RADS 4 and 5 lesions. Although PHI is clinically useful, it remains imperfect; S2,3PSA% provides complementary information by capturing PC-specific glycan alterations. Their combined use can enhance diagnostic accuracy and support more refined biopsy decision-making. By contrast, the csPC detection rate remained low for PI-RADS 3 lesions (16%), which highlights the challenge in managing equivocal MRI findings, even when combined with biomarkers.

Established MRI-adapted risk calculators such as the ERSPC and PHI models [17], [18], [27], [29], [33], [34] are widely used to guide biopsy decisions. Our findings suggest that addition of S2,3PSA%—a glycan-informed PSA metric—to PI-RADS and PHI provides complementary biological information and improves discrimination beyond existing calculators. Furthermore, emerging evidence indicates that S2,3PSA% may also assist in monitoring men on active surveillance by helping to predict reclassification [35], which offers potential to enhance both biopsy selection and longitudinal disease management.

In clinical practice, men with PI-RADS ≥4 lesions are typically biopsied regardless of biomarker results, while S2,3PSA% and PHI assist in decision-making for PI-RADS 3 or indeterminate lesions. However, no single marker—including imaging—can detect all csPC with complete accuracy. Diagnostic accuracy and practicality must be balanced; therefore, biopsy decisions should rely on shared decision-making. Our integrated model supports this process but requires further refinement and external validation.

Our study has several limitations. The study was retrospective in nature, which introduces potential selection and information biases that cannot be fully eliminated. Although data were prospectively collected and consecutively analyzed, prospective multicenter validation will be necessary to confirm the generalizability and clinical applicability of our findings. MRI scans were performed across institutions using different scanners, mostly 1.5-T systems. This heterogeneity may have affected lesion visibility and PI-RADS grading, which might introduce variability in model performance. The study included only men who underwent MRI-US fusion biopsy, and 122 men with low risk chose surveillance, which possibly introduced selection bias and thus overestimation of biomarker performance. The study included referred patients with MRI-visible lesions, with PHI and S2,3PSA% measured under a research protocol. Thus, selection bias towards men with higher PC risk cannot be excluded. Because the study included only patients who underwent MRI-US fusion targeted biopsy, selection bias towards men with PI-RADS 3–5 lesions cannot be excluded. Although PI-RADS evaluation was performed primarily by a single radiologist to ensure consistency, inter-reader variability in MRI interpretation is well recognized across institutions and could affect reproducibility. Although MRI-US fusion biopsy is the reference standard, it may misclassify lesions, and follow-up confirmation was unavailable. Although internal validation via bootstrapping confirmed some overfitting, external validation using independent, multicenter data sets is necessary to ensure the generalizability of our findings. To address these issues, future multi-institutional, prospective studies are needed to externally validate risk score–based approaches and confirm their clinical utility for biopsy decision-making.

## 5 Conclusions

Our results suggest that integrated assessment of S2,3PSA%, PHI, and MRI improves prediction of csPC. Future research should validate this model externally in multi-institutional cohorts, test the reproducibility of S2,3PSA% measurement, and evaluate the clinical utility of the model in prospective decision-making studies.

  ***Author contributions*:** Shingo Hatakeyama had full access to all the data in the study and takes responsibility for the integrity of the data and the accuracy of the data analysis.

  *Study concept and design*: Hatakeyama, Yoneyama, Oishi.

*Acquisition of data*: All authors.

*Analysis and interpretation of data*: Hatakeyama.

*Drafting of the manuscript*: Hatakeyama, Takuya Oishi.

*Critical revision of the manuscript for important intellectual content*: All authors.

*Statistical analysis*: Hatakeyama, Oishi.

Obtaining funding: Hatakeyama.

*Administrative, technical, or material support*: All authors.

*Supervision*: Ohyama.

*Other*: None.

  ***Financial disclosures*:** Shingo Hatakeyama certifies that all conflicts of interest, including specific financial interests and relationships and affiliations relevant to the subject matter or materials discussed in the manuscript (eg, employment/affiliation, grants or funding, consultancies, honoraria, stock ownership or options, expert testimony, royalties, or patents filed, received, or pending), are the following: Shingo Hatakeyama reports honoraria from Janssen Pharmaceutical, Astellas Pharma, AstraZeneca, Ono Pharmaceutical, Bayer, Pfizer, Bristol-Myers Squibb, Merck Biopharma, Kaneka Corporation, FUJIFILM Wako Pure Chemical Corporation, and Nipro Corporation. The remaining authors have nothing to disclose.

  ***Funding/Support and role of the sponsor*:** This work was supported by the 10.13039/501100001691Japan Society for the Promotion of Science KAKENHI (grant 25K02769 to Shingo Hatakeyama, grant 24K12501 to Tohru Yoneyama).

  ***Acknowledgments*:** We thank all the study participants and Satoko Sakamoto, Mitsuharu Miyadate, and Yukie Nishizawa for their help with the data collection and analysis.

  ***Data sharing statement*:** The data that support the findings of this study are available from the corresponding author on reasonable request.

  ***Ethics statement*:** This retrospective, multicenter study adhered to the tenets of the Declaration of Helsinki and was approved by the ethics committee of the Hirosaki University School of Medicine (2019-099-3 and 2022-140-2). All participants provided written informed consent to participate in this study.
